# Covered versus uncovered self-expandable metallic stents for palliation of malignant gastric outlet obstruction: a systematic review and meta-analysis

**DOI:** 10.1186/1471-230X-14-170

**Published:** 2014-09-30

**Authors:** Ya-min Pan, Ji Pan, Li-kun Guo, Min Qiu, Jia-jun Zhang

**Affiliations:** Department of Endoscopy, Shu-guang Hospital Affiliated to Shanghai University of Traditional Chinese Medicine, 201203 Shanghai, China

**Keywords:** Covered SEMSs, Uncovered SEMSs, Gastric outlet obstruction, Meta-analysis

## Abstract

**Background:**

Self-expandable metallic stents (SEMSs) are widely used for palliation of malignant gastric outlet obstruction (GOO). There are two types of SEMS, covered and uncovered, each with its own advantages and disadvantages. We aimed to compare the efficacy and safety between uncovered and covered SEMSs in the palliation of malignant gastric outlet obstruction.

**Methods:**

Databases including PubMed, EMBASE, the Cochrane Library, the Science Citation Index and momentous meeting abstracts were searched and evaluated by two reviewers independently.

**Results:**

Nine trials involving 849 patients were analyzed. Meta-analysis showed there was no significant difference in technical success rate (RR 1.0, 95% CI [0.98, 1.01]), clinical success rate (RR 1.04, 95% CI [0.98, 1.11]), post-stenting dysphagia score (WMD −0.01, 95% CI [−0.52, 0.50]), stent patency (WMD −0.31, 95% CI [−1.73, 1.11]), overall complications (RR 1.07, 95% CI [0.87, 1.32]) and reintervention rate (RR 1.30, 95% CI [0.92, 1.83]) between covered and uncovered SEMSs group. However, covered SEMSs were associated with higher migration rate (RR 3.48, 95% CI [2.16, 5.62], P < 0.00001) and lower obstruction rate (RR 0.42, 95% CI [0.24, 0.73], P = 0.002).

**Conclusions:**

In the palliative treatment of malignant gastric outlet obstruction, both covered and uncovered SEMSs are safely and effective. Covered stents can reduce the risk of restenosis, whereas uncovered stents are effective in decreasing stent migration.

## Background

Malignant gastric outlet obstruction (GOO) is recognized as a complication of advanced malignant disease in the upper gastrointestinal tract, which usually include distal gastric cancer, periampullary carcinoma, lymphoma and metastases to the duodenum [1, 2]. GOO always leads to intractable vomiting, nausea, abdominal discomfort and poor oral food intake, which diminish quality of life. Compared with palliative gastrojejunostomy or other surgical procedures, self-expandable metallic stents (SEMSs) can rapidly relieve obstructive symptoms with fewer complications and mortality [3, 4]. There are two types of SEMSs, covered and uncovered type; both of them are widely used for palliation of GOO [5–7], each with its own advantages and disadvantages.

To the best of our knowledge, a systematic review [8] on this topic has been published. More recently, additional studies have been published and some conflicting results have emerged. Therefore, we believe an updated systemic review and meta-analysis is required to evaluate the efficacy and safety between uncovered and covered SEMSs for palliation of malignant gastric outlet obstruction.

## Methods

### Study identification and eligibility criteria

This study has been approved by ethics committee of Shu-guang Hospital Affiliated to Shanghai University of Traditional Chinese Medicine. A comprehensive literature search was done to identify all relevant studies that compared covered stents with uncovered stents in the palliation of malignant gastric outlet obstruction. The PubMed, EMBASE, the Cochrane Library and the Science Citation Index were searched systematically for all articles published up to Dec.2013, without language restriction, which included the following terms in their titles, abstracts, or keywords lists: “gastroduodenal obstruction”, “covered stent”, “uncovered stent”, “malignant gastric outlet obstruction”. The references in retrieved articles were also screened manually. The abstracts of United European Gastroenterology week (UEGW) and Digestive Disease Week (DDW) were also searched systematically.

The inclusion criteria were as follows: (1) RCTs and nonrandomized prospective and retrospective studies; (2) analyses of both uncovered stents and covered stents; (3) patients were diagnosed malignant gastric outlet obstruction; (4) outcome measures included technical and clinical success, overall complications, stent patency and reintervention rate; (5) when multiple articles published by the same team from the same institute within the same study interval were identified, only the latest or the most detailed and informative article, or the one with the best quality in methodology, was included. Commentaries, case reports, reviews, or guidelines were excluded.

### Data extraction

Two reviewers (YM.P and J.P) abstracted data independently and reached consensus on all items. Data were extracted on: first author; year of publication; study interval; number of patients; age and sex; study design, stent characteristics; technical success rate; clinical success rate; stent obstruction; stent migration; overall complications; reintervention rate; stent patency and GOO scoring system (GOOSS).

### Assessment of methodological quality

The quality of all studies was assessed by using the Newcastle-Ottawa Scale with some modifications to match the needs for this meta-analysis [9]. The quality of the studies was evaluated by examining three items: patient selection, comparability of study groups, and assessment of outcome. Studies achieving five or more stars were considered high quality. Methodological quality assessment was independently carried out by two of the authors. Any disagreement was resolved by consensus.

### Statistical analysis

Statistical manipulation was performed with Review Manager Software (Version 5.1, Windows, Nordic Cochrane Center, Copenhagen, Denmark). Values for analysis were extracted from published reports or calculated from crude data. For summary statistics in meta-analyses, the Relative Risk (RR) is recommended for dichotomous data, and the Weighted Mean Difference (WMD) is recommended for continuous data. Pooled estimates were presented with a 95% confidence interval. P < 0.05 was considered significant. A sensitivity analysis [10] determined how the results would be influenced if one study was removed from the analysis for each outcome; this indicates the extent to which the results are (or are not) robust to assumptions and decisions that were made when carrying out the synthesis. Because of the anticipated clinical heterogeneity across the included studies, we decided to use only the random effect model before pooling data. This adjusts for variability of results among studies and provides a more conservative estimate of an effect by using a wider confidence interval [11].

## Results

### Identification of eligible studies

**Figure 1:**
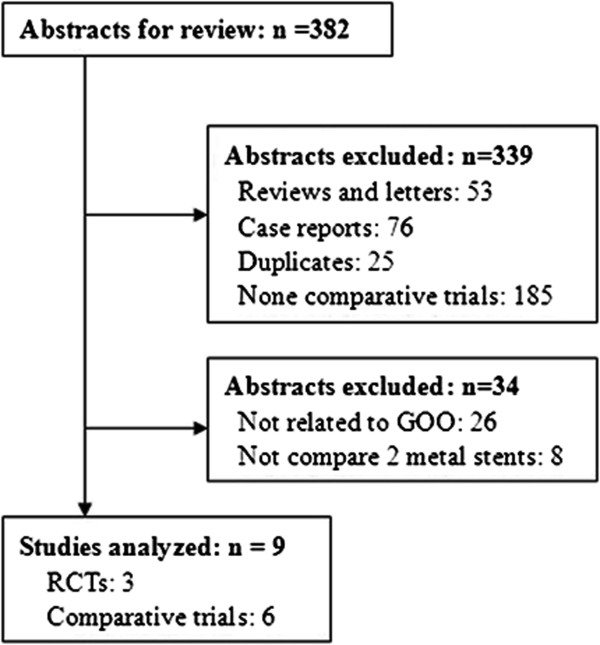
**Flow diagram of trials selection.** GOO, gastric outlet obstruction; RCT, randomized clinical trial.

The literature search yielded 382 abstracts for review. Finally, 9 trials [5–7, 12–17] were included (Figure 1). 3 trials [5, 14, 16] were randomized clinical trials, 5 trials [6, 7, 12, 15, 17] were retrospective studies and one report [13] was prospective study. The combined studies enrolled 849 patients, of whom 380 underwent covered stents placement and 469 with uncovered stents. The characteristics of included trials were listed in Table 1. The quality assessment and scores are summarized in Table 1. Seven studies scored five or more stars on the modified Newcastle Ottawa Scale [5, 12–17]. Characteristics of stents were not consistent in all studies (Table 2). More than two types of stents were used in 3 trials [14, 15, 17]. The main composition of covered materials is polytetrafluoroethylene membrane or polyurethane membrane.

**Table 1:** **Characteristics of the included studies**

Study author	Year	Design	Study interval (y)	Patients (n) covered/uncovered	Age (M, y) covered/uncovered	Female (n, %)	Disease diagnosis (n)	Matching	Quality score (*)
Yu Kyung Cho [5]	2004	RCT	NR	13/12	65.0^§^	12(48%)	GC(25)	1,2,3	*****
Jong Pil Im [6]	2008	R	2005-2006	24/18	60.7^§^	7(16%)	GC(26)/PC(12) /Others(13)	NR	****
Seungmin Bang [7]	2008	R	1998-2003	53/79	58.0/59.0	40(30%)	GC(109)/PC(13) /Others(12)	NR	***
Iruru Maetani [12]	2009	R	1998-2006	29/31	70.6/72.2	27(45%)	GC(28)/PC(20)/ Others(12)	1,2,3,4,5	*****
Kee Myung Lee [13]	2009	P	1998-2007	70/84	67.2/63.3	48(31%)	GC(122)/PC(19) /Others(13)	1,2,3,5	*****
Chan Gyoo Kim [14]	2010	RCT	2003-2007	40/40	58.0/57.0	17(21%)	GC(80)	1,2,3,4,5	******
Chan Ik Park [15]	2012	R	2006-2011	96/128	64.0/65.0	15(17%)	GC(224)	1,2,5	*****
Iruru Maetani [16]	2013	RCT	2007-2010	31/31	69.4/68.1	32(52%)	GC(27)/PC(26) /Others(9)	1,2,3,4,5	******
Sang Myung Woo [17]	2013	R	2003-2010	24/46	62.0/61.0	30(43%)	PC(46) /Others(24)	1,2,3,4,5	*****

R, retrospective; P, prospective; RCT, randomized control trial; NR, not reported.

^§^, mean age of all patients; (*), star rating (max 9).

Matching: 1, age; 2, sex; 3, diagnosis; 4, site of obstruction; 5, previous treatment.

GC, gastric cancer; PC, pancreatic cancer.

Others, including gallbladder cancer, bile duct cancer, ampullary cancer, duodenal cancer and metastasis.

**Table 2:** **Stent characteristics of the included studies**

Study/author	Group	Stent type	Stent material	Stent diameter (mm)	Stent length (cm)	Covered material
Yu Kyung Cho	Covered	NR	Nickel-Titanium	NR	NR	NR
	Uncovered		Nickel-Titanium			
Jong Pil Im	Covered	NR		18	9/11	
	Uncovered		NR	18	11/12/16	NR
Seungmin Bang	Covered	Niti-S	NR	20/22	6-15	
	Uncovered	Niti-S		20/22	6-15	PU
Iruru Maetani	Covered	Ultraflex	NR	18/23	10/12/15	
	Uncovered	Ultraflex		18/23	10/12/15	PTFE
Kee Myung Lee	Covered	Niti-S	Nitinol	18	6/8/10	
	Uncovered	Niti-S	Nickel-Titanium	18	6/8/10	PU
Chan Gyoo Kim	Covered	Niti-S	Nitinol	18/20	8/10/12	
	Uncovered	Wallstent/Wallflex	Elgiloy/Nitinol	20/22	6/9/12	PTFE
Chan Ik Park	Covered	Niti-s	Nitinol	NR	6-16	
	Uncovered	Wallstent/Hanaro	Elgiloy/Nitinol		6-16	PU
Iruru Maetani	Covered	Niti-S	Nitinol	20		
	Uncovered	Comvi	Nitinol	20	NR	PTFE
Sang Myung Woo	Covered	Niti-s/Bona		18-22	4-12	
	Uncovered	Niti-s/Bona/Wallflex	NR	18-22	4-12	NR

PTEF, polytetrafluoroethylene; PU, polyurethane.

Niti-S (Taewoon Inc. South Korea); Bona (Standard SciTech Inc. Seoul, Korea).

Hanaro (M.I. Tech. Seoul, Korea).

Wallflex (Boston Scientific, USA); Wallstent (Boston Scientific, USA).

Ultraflex (Boston Scientific, USA).

### Technical and clinical success rate

**Figure 2:**
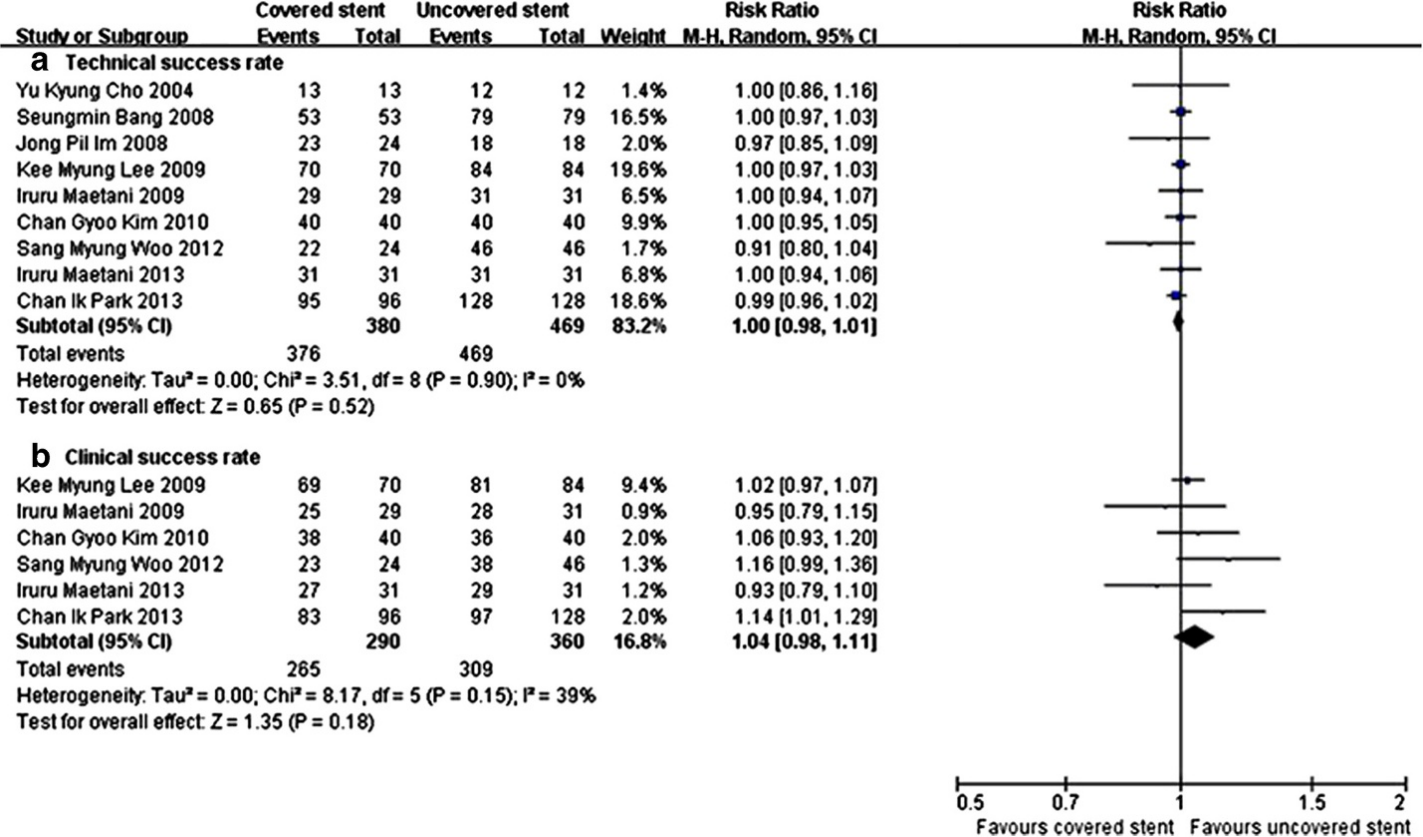
**Meta-analysis showed there was no significant difference in technical success rate (a) and clinical success rate (b) between covered and uncovered stents group.**

**Figure 3:**
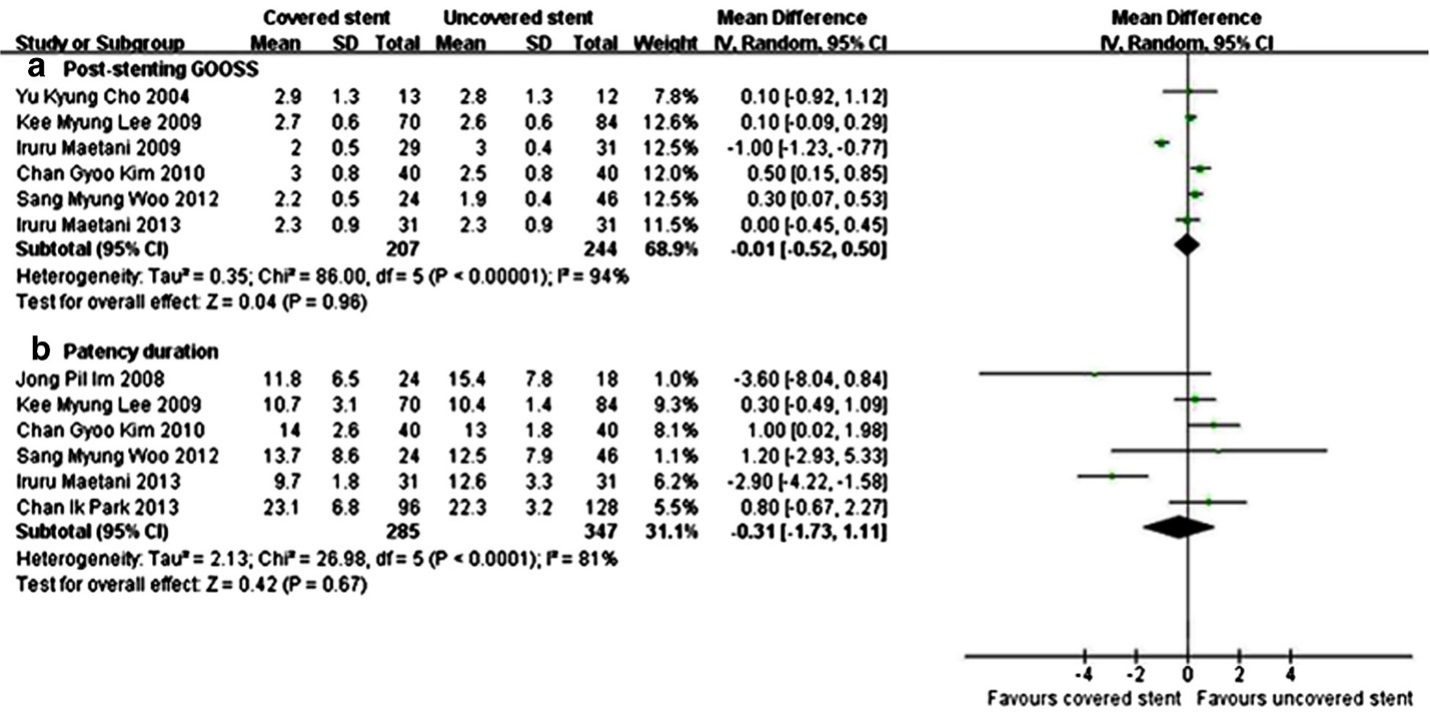
**Meta-analysis showed there was no significant difference in post-stenting GOOSS (a) and stent patency (b) between covered and uncovered stents group.**

All trials assessed the technical success rate related to stenting procedure, and 6 trials [12–17] assessed the clinical success rate. Meta-analysis showed that there was no significant difference in technical success rate (RR 1.00, 95% CI [0.98, 1.01]) (Figure 2a) and clinical success rate (RR 1.04, 95% CI [0.98, 1.11]) (Figure 2b). The post-stenting GOOSS was recorded in 6 trials [5, 12–16], there was no significant difference between covered and uncovered stents group (P = 0.96) (Figure 3a).

### Stent patency

The stent patency was reported in 6 studies [6, 13–17]. The median patency duration was 9 to 23 weeks with covered stents and 10 to 22 weeks with uncovered stents respectively. Meta-analysis showed there was no significant difference between covered and uncovered stents group (WMD −0.31, 95% CI [−1.73, 1.11]) (Figure 3b). Chemotherapy after endoscopic stenting was reported in 6 studies, totally 42.7% (270/632) patients underwent chemotherapy. All studies reported that palliative chemotherapy was not associated with stent patency.

### Complications and reintervention

**Figure 4:**
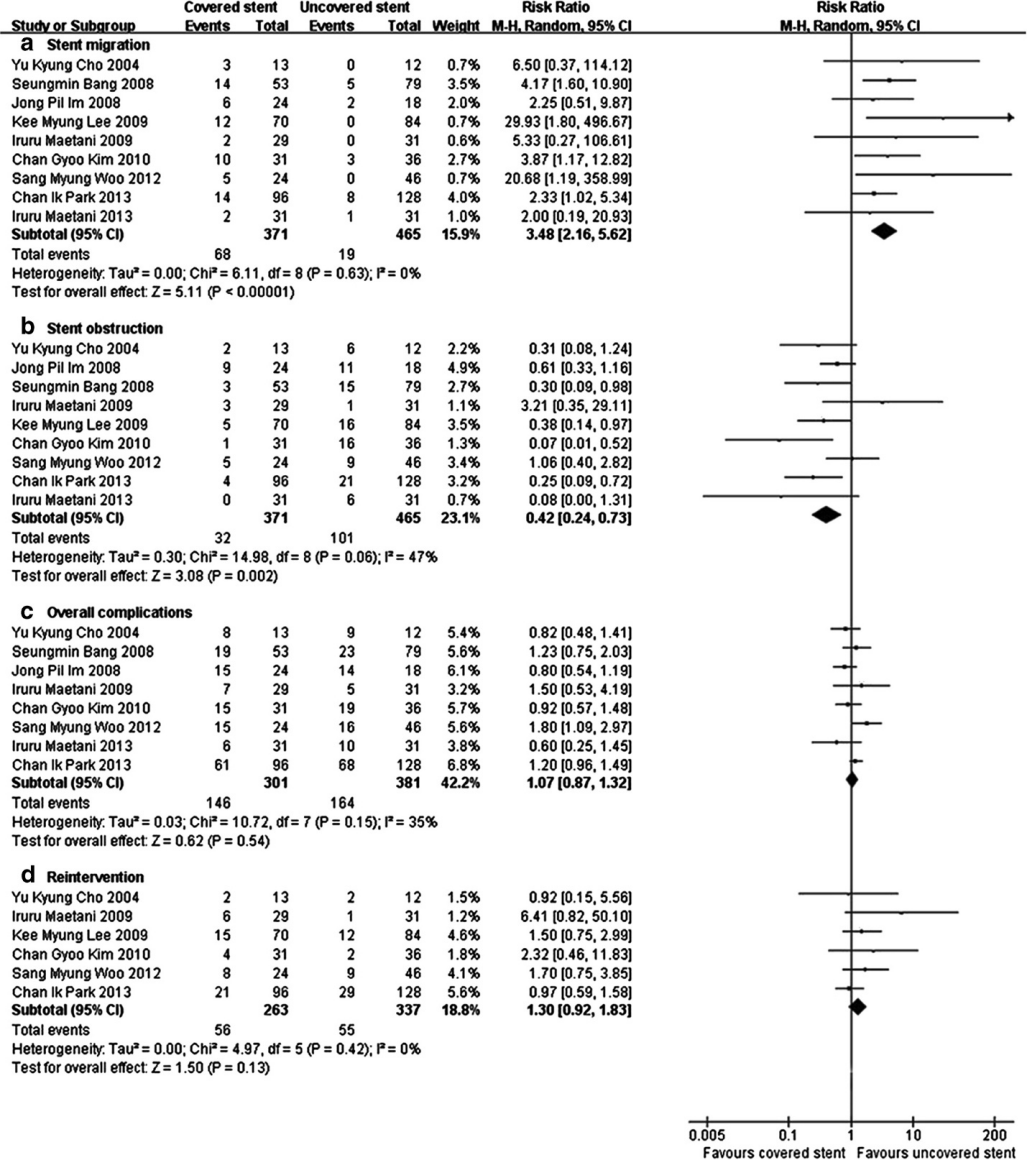
**Complications and reintervention between covered and uncovered metal stents group.** Meta-analysis showed there was no significant difference in overall complications **(c)**. However, covered stents were associated with higher stent migration **(a)** and less restenosis **(b)** compared to uncovered stents. There was no significant difference in reintervention rate between two groups **(d)**.

Meta-analysis showed there were no significant difference in overall complications between covered and uncovered stents group (P = 0.54) (Figure 4c). However, covered stents were associated with higher stent migration (RR 3.48, 95% CI [2.16, 5.62]) (Figure 4a), and uncovered stents were associated with higher stent obstruction (RR 0.42, 95% CI [0.24, 0.73]) (Figure 4b) in subgroup analysis. Reintervention for stent-related complications was reported in 6 studies [5, 12–15, 17]. Meta-analysis showed there was no significant difference in reintervention rate between two groups (P = 0.13) (Figure 4d).

### Sensitivity analysis

The results of sensitivity analysis are listed in Table 3. When high quality studies were analyzed (Star ≥ 5), meta-analysis showed there was no significant difference in technical success rate, clinical success rate, stent patency, overall complications, Post-stenting GOOSS and reinterventioon rate between two groups. Covered stents were associated with higher stent migration rate (RR 3.49, 95% CI [1.92, 6.32]) and lower stent obstruction rate (RR 0.39, 95% CI [0.18, 0.84]) compared to uncovered stents. In the studies containing N ≥ 70 patients, meta-analysis showed the same results. Data were also analyzed by random effects models. Sensitivity analysis showed that the results were robust.

**Table 3:** **Sensitivity analysis**

Primary outcome	No. studies	No. patients	Rate (%) (Covered⁄Uncovered)	RR⁄WMD (95% CI)	P-value
**High quality studies(Star** ≥ **5)**					
Technical success rate	7	675	99.0/100	0.99 [0.98, 1.01]	0.51
Clinical success rate	6	650	91.4/85.8	1.04 [0.98, 1.11]	0.18
Post-stenting GOOSS	6	451	-	−0.01 [−0.52, 0.50]*	0.96
Stent pantency	5	590	-	−0.05 [−1.50, 1.39]*	0.94
Stent migration	7	662	16.3/3.3	3.49 [1.92, 6.32]	<0.0001
Stent obstruction	7	662	6.8/20.4	0.39 [0.18, 0.84]	0.02
Overall complications	6	508	50.0/44.7	1.11 [0.86, 1.44]	0.43
Reintervention rate	6	600	21.3/16.3	1.30 [0.92, 1.83]	0.13
**Studies containing** ≥ **70 patients**					
Technical success rate	5	660	98.9/100	1.00 [0.98, 1.01]	0.53
Clinical success rate	4	528	92.6/84.6	1.08 [0.99, 1.18]	0.08
Post-stenting GOOSS	3	304	-	0.26 [−0.05, 0.48]*	0.06
Stent pantency	4	528	-	0.62 [−0.06, 1.18]*	0.07
Stent migration	5	647	20.1/4.3	4.00 [2.05, 7.80]	<0.0001
Stent obstruction	5	647	6.6/20.6	0.35 [0.17, 0.74]	0.006
Overall complications	4	493	53.9/43.6	1.22 [0.99, 1.51]	0.06
Reintervention rate	4	515	21.7/17.7	1.25 [0.88, 1.78]	0.21

R, retrospective trial; P, prospective trial; RCT, randomized control trial.

*, WMD (95%CI).

GOOSS, gastric outlet obstruction scoring system.

## Discussion

Malignant gastric outlet obstruction without effective intervention would result in progressive deterioration and death [3]. Surgical bypass has been the standard treatment, but it is associated with significantly high morbidity and mortality. Endoscopic stenting is an alternative treatment, which palliates malignant obstruction with lower morbidity and mortality [4]. There are two types SEMSs widely used in clinical, covered and uncovered. Which is better? Maetani et al. [6] reported covered stents were associated with more frequent need for reintervention than uncovered stents. Kim et al. [14] reported both covered and uncovered SEMSs were effective and safe in treatment of patients. Recently, a systematic review [8] on this topic has been published. However, only two RCTs related to GOO were included for analysis in this systematic review, the number of included patients may be too small to make effective statistic analysis. More recently, additional studies have been published and some conflicting results have emerged. Therefore, we believe an updated systemic review and meta-analysis is required to evaluate the efficacy and safety between uncovered and covered SEMSs.

In this meta-analysis, there was no significantly difference between uncovered and covered stents in technical and clinical success rates. Endoscopic stenting was deployed through a guide wire, when the guide wire could not pass the stricture, and then the stent could not be deployed successfully. So the success of stenting is affected by the degree of narrowing and tortuosity of the stricture rather than the difference between the types of stent used.

The GOOSS was used to evaluate the severity of obstructive symptom, the GOOSS assigns a point score depending on the patient’s level of oral intake (no oral intake, 0; liquids only, 1; soft solids, 2; low-residue or full diet, 3) [18]. Most of patients could not intake any fluid before stenting, though most included studies were retrospective, the patients’ characteristics were similar in baseline. There was significant difference in GOOSS pre-stenting and post-stenting, but there was no difference between covered and uncovered stents after stenting. These mean that both stents have similar effect in palliation of malignant obstruction.

Stent patency is an important factor in QOL of patients. Meta-analysis showed there was no significant difference in stent patency between two groups. The main cause influencing patency was stent migration in covered stent group and stent obstruction in uncovered stent group respectively. The covered stent was associated with advantage of preventing tumor ingrowth, but this advantage was offsetted by high migration. Chemotherapy after stent placement could be independently associated with prolonged stent patency, because chemotherapy may stabilize or decrease tumor burden and thereby decrease malignant ingrowth or overgrowth. Some studies [19, 20] reported chemotherapy after stent placement contributed to longer durations of stent patency in gastric cancer patients. However, we could not find any association of stent patency with palliative chemotherapy after stent placement in included studies. Further prospective randomized trials are needed to determine the role of chemotherapy.

Complications included stent obstruction, stent migration, bleeding, stent fracture, perforation and others, the most complications were stent obstruction and migration. There was no difference in overall complications between the covered stent group and uncovered stent group. One study [21] has shown that covered stents placed in the biliary tract prevent tumor ingrowth without increasing migration frequency. However, in subgroup analysis, our meta-analysis showed that stent migration was more frequent in the covered stent group, which is maybe the expansion force of the covered stent is transferred to the intestinal wall through the covering membrane rather than through the wire mesh, and the friction between stent and tumor might not be enough to keep the stent stationary. The cause of stent obstruction included tumor ingrowth and overgrowth. Meta-analysis showed uncovered stents were associated with higher stent obstruction compared to covered stents, because uncovered stents are often associated with tumor ingrowth through the stent mesh. 3 trials reported the tumor overgrowth rate; there was no significant difference between two groups. When stent migration or stent obstruction occurred, endoscopic or surgical interventions should be taken. Though 3 trials reported uncovered stent was associated with lower re-intervention rate, meta-analysis showed there was no significant difference between covered and uncovered stents.

Several limitations of the present study need to be considered. First, there was significant heterogeneity for main outcomes. The source of heterogeneity may include the different publishing time of studies, the study design (6 none-RCT studies), the selection criteria, the characteristic of patients and stents. Though the data was treated with random effect models, there was still some influence to final results. Second, included studies were associated with small sample sizes, different levels of the intervention, different follow-up duration; those could also influence the results. Third, there were many different causes of GOO, which including gastric cancer, pancreatic cancer and others. The different characteristic of diseases might lead to different stent patency and complications. Fourth, the selective reporting of studies with positive results may result in overestimation of technical, clinical success rate and stent patency, and cause some bias to our meta-analysis.

## Conclusions

Both covered and uncovered SEMSs are technically feasible and effective in the palliative treatment of malignant gastric outlet obstruction. Meta-analysis showed there was no significant difference in stent patency, overall complications and reintervention; whereas in subgroup analysis, stent obstruction was more frequent with covered stents, and stent migration occurred more frequently with uncovered stents. The retrospective nature of these reports and their small sample sizes suggest that prospective controlled trials with large sample sizes are required to confirm the results of the current meta-analysis.

## Consent

Written informed consent was obtained from the patient for the publication of this report and any accompanying images.
